# Commentary: Solithromycin mitigates *Prevotella intermedia*–induced methicillin-resistant *Staphylococcus aureus* ventilator-associated pneumonia by enhancing alveolar macrophage function

**DOI:** 10.3389/fcimb.2026.1825239

**Published:** 2026-05-12

**Authors:** Kai-Li Mao, Liu-Cheng Li, Lian-Di Kan, Qiu Jiang

**Affiliations:** 1 Department of Pharmacy, The Quzhou Affiliated Hospital of Wenzhou Medical University, Quzhou People’s Hospital, Quzhou, China; 2 Department of Pharmacy, Sir Run Run Shaw Hospital, Zhejiang University School of Medicine, Hangzhou, China

**Keywords:** immunomodulation, macrophage reprogramming, osteomyelitis, solithromycin, translational roadmap

This Editorial introduces the Research Topic “Oral Microbes and Host”. In this context, we highlight the work by Fukushima et al. (2026) as a prime example of how mechanistic insights from related fields (pneumonia) can be translated into testable hypotheses for osteomyelitis. Bone infections encompass osteomyelitis, periprosthetic joint infections, and infected bone defects. With aging populations and increasing orthopedic procedures, infection rates for open fractures range from 5%-33%, and joint replacement infections affect 1%-4% of procedures. Treatment failure rates remain high at 10%-40% (Qin et al., 2024). Staphylococcus aureus accounts for over 70% of cases. It employs multiple evasion mechanisms: intracellular survival within osteoblasts and macrophages, biofilm formation on implants, occupation of osteocyte canalicular networks, and formation of staphylococcal abscess communities protected by fibrin pseudocapsules. Clinically, the management of infectious bone diseases is further complicated by the avascular nature of necrotic bone tissue, which limits antibiotic penetration, and the formation of sluggish biofilms on implanted hardware. These mechanisms render conventional antibiotics ineffective and contribute to 20-30% recurrence rates. The host acute phase response is critical for defense, but dysregulated inflammation can drive severe illness (Hunter et al., 2026). C-reactive protein trends provide prognostic information, with sustained elevation associated with worse outcomes. Genetic factors influence susceptibility, with very young children, specific ethnic groups, and immunocompromised patients facing higher risk (Yap et al., 2023; Hunter et al., 2026). Diagnosis requires integration of histopathology, microbiology, and advanced imaging (18F-FDG PET/CT, labeled leukocyte scans). Treatment involves surgical debridement combined with systemic and local antibiotic delivery. The coupling of infection control with bone regeneration in infected defects remains a major therapeutic gap.

Fukushima et al. (2026) investigated whether solithromycin (SOL), a fourth-generation macrolide, could mitigate Prevotella intermedia culture supernatant-exacerbated MRSA ventilator-associated pneumonia. The study employed murine models, alveolar macrophage-like cells, transcriptomics (RT-qPCR, RNA-seq), and flow cytometry. P. intermedia supernatant significantly reduced survival in MRSA-infected mice, an effect completely abrogated by SOL treatment. SOL-treated supernatant reduced lung MRSA burden, promoted macrophage recruitment, and upregulated Ccr2 expression. RNA-seq revealed that SOL upregulated macrophage antimicrobial genes (Clec4e, Cybb, Irgm1) and attenuated excessive inflammation. *In vitro*, SOL-pretreated macrophages exhibited enhanced bactericidal activity and Tnf-α upregulation. SOL’s protective effects operate through: (1) inhibiting bacterial protein synthesis in P. intermedia, reducing virulence factor production; (2) enhancing macrophage recruitment and function; (3) modulating inflammatory pathways; and (4) directly activating macrophages even at sub-MIC concentrations.

The study fills a critical knowledge gap in the “Oral Microbes and Host” topic by demonstrating that Prevotella intermedia culture supernatant exacerbates MRSA ventilator-associated pneumonia (VAP) through impairing alveolar macrophage function and promoting excessive neutrophilic inflammation, while SOL, even at sub-MIC levels, restores host defense by upregulating macrophage phagocytosis, Ccr2, Tnf-α, and interferon-related pathways (Fukushima et al., 2026). This extends prior work by showing that SOL enhances, rather than suppresses host defense, primarily through reprogramming macrophage function.

While the Fukushima study focused on pulmonary infection, the mechanistic principles, particularly the enhancement of intracellular killing genes (Irgm1, Cybb) and the modulation of excessive inflammation, are directly translatable to the osteoimmune environment. Bone homeostasis relies on a delicate balance between osteoblasts and osteoclasts, the latter of which are derived from the macrophage lineage. Given that S. aureus persists intracellularly within both osteoblasts and macrophages in bone infections, SOL’s demonstrated ability to reprogram macrophage antimicrobial function provides a strong rationale for testing its efficacy in orthopedic contexts. Specifically, the following observations are hypothesis-generating for osteomyelitis research: macrophage modulation (dual roles in clearance and bone destruction), intracellular bacterial persistence, polymicrobial interactions, and immunomodulatory therapy potential. Whether SOL’s effects are restricted to macrophages or also affect osteoblast intracellular killing remains unknown. Additionally, optimal delivery strategies (e.g., local scaffolds), synergy with conventional antibiotics, and long-term effects on bone regeneration require direct investigation in bone infection models.

Beyond the questions raised above, several more fundamental constraints further limit generalization from pneumonia to osteomyelitis. First, tissue microenvironmental differences present a fundamental barrier: the alveolar space is immunologically unique, with abundant resident macrophages and surfactant proteins, whereas bone is a hypoxic, nutrient-limited environment where osteocytes reside within lacunae and pathogens form biofilms on avascular necrotic tissue or implant surfaces. Second, the pathogen context differs substantially, the Fukushima model examined planktonic MRSA growth exacerbated by P. intermedia supernatant, while osteomyelitis is predominantly driven by biofilm-encased S. aureus, which exhibits markedly different antibiotic susceptibility and immune evasion profiles. Third, the original pneumonia model did not include osteoblasts or osteoclasts, leaving their responses to SOL entirely unexplored. Fourth, pharmacokinetic considerations limit extrapolation: SOL achieved efficacy only via intraperitoneal administration in the pneumonia model, with oral delivery ineffective; achievable SOL concentrations in bone tissue, particularly within avascular sequestra or biofilms, remain uncharacterized. Fifth, polymicrobial interactions in osteomyelitis are more complex, often involving anaerobes (e.g., Finegoldia magna, Peptostreptococcus spp.) and facultative organisms synergistically affecting virulence and antibiotic tolerance, interactions not captured by the two-pathogen model used.

This Editorial does not claim SOL efficacy in osteomyelitis but translates mechanistic insights from pneumonia into testable hypotheses: intracellular killing in osteoblasts, synergy with antibiotics, and scaffold-based delivery. Addressing these will determine whether SOL warrants clinical investigation in infectious bone disease.
